# Necessity for subsequent surgery in women of child-bearing age with positive margins after conization

**DOI:** 10.1186/s12905-021-01329-x

**Published:** 2021-05-07

**Authors:** Xinmei Wang, Juan Xu, Yang Gao, Pengpeng Qu

**Affiliations:** 1 Clinical College of Central Gynecology and Obstetrics, Tianjin Medical University, Tianjin, 300070 China; 2 Department of Gynecological Oncology, Tianjin Central Hospital of Gynecology and Obstetrics, Tianjin, 300100 China

**Keywords:** Cervical intraepithelial neoplasia (CIN), Women of child-bearing age, Cold knife conization (CKC), Positive margins, Residual lesions, High-risk HPV

## Abstract

**Background:**

20–25% of women with high-grade cervical intraepithelial neoplasias (HSIL) have residual lesions after conization. The state of the margin is generally considered to be a risk factor for recurrence or persistent lesions. Predictors of positive margins and residual lesions need to be identified. A design for postoperative management and avoidance of overtreatment needs to be provided, especially for women of child-bearing age.

**Methods:**

This study was a retrospective analysis of 1309 women of child-bearing age with HSIL, who underwent cold knife conization (CKC). Age, gravidity, parity, human papillomavirus (HPV) species, cytology, transformation zone type, results of endocervical curettage (ECC), quadrant involvement, glandular involvement, and Cervical Intraepithelial Neoplasia (CIN) grade were analyzed. Among those with positive margins, 245 patients underwent secondary surgery within three months, including CKC, a loop electrosurgical excision procedure, and total hysterectomy. Risk factors for positive margins and residual lesions were assessed.

**Results:**

There was no significant difference in age, gravidity, parity, glandular involvement, and CIN grade between the two groups (*P* > 0.3). There was a significant difference in HPV species, cytology, ECC results, and quadrant involvement (*P* < 0.002). Multivariate analysis showed a major cytology abnormality, high-risk HPV infection, type III transformation zone, positive ECC result, and multiple quadrant involvement were independent risk factors for positive margins and residual lesions (*P* < 0.02). Age > 35 years was also a risk factor for residual lesions (*P* < 0.03).

**Conclusion:**

High-risk women should be treated appropriately considering fertility. Patients with positive margins should be managed uniquely. Surgery for women without fertility may be appropriate. Close follow-up is necessary for women who have fertility requirements or are unwilling to undergo subsequent surgery if they have no risk factors, especially women < 35 years.

## Background

In 2015, the American Society of Colposcopy and Cervical Pathology (ASCCP) recommended that one of the standard treatment options for cervical squamous intraepithelial lesions (SIL), especially HSIL [including CIN3 and part of CIN2], was cervical conization [1], including LEEP and CKC. However, these two types of conization have a common limitation; that is, 20–25% of women have residual lesions after an operation [2].The state of the margin is generally considered to be a risk factor for recurrence or persistent CIN [3, 4].A comprehensive meta-analysis by Debeaudrap revealed that the rate of positive margins after conization was about 23.0% (8091/35,109) [5].

With improvement of people’s awareness of health care and advances in detection technology, the age of onset for this disease is becoming increasingly younger. Furthermore, due to changes in China’s family planning policy in recent years, an increasing number of women of child-bearing age have fertility requirements. Although hysterectomy is the definitive therapy for women with positive margins who have no reproductive requirements, cervical conization is considered an acceptable alternative in women who desire fertility preservation. There have been conflicting reports regarding recurrence rates and residual disease in women undergoing cervical conization for CIN [2, 4, 5]. Moreover, secondary conization can affect conception and lead to adverse pregnancy outcomes [6, 7]. Therefore, the ideal management of women of child-bearing age with positive margins remains controversial.

In order to get the management model of women of child-bearing age with positive margins, the clinicopathological data of 1309 women of child-bearing age with high-grade CIN (including CIN3 and CIN2) and 245 cases with positive margins who underwent subsequent surgery within three months was analyzed, and the risk factors of positive margins and residual lesions were explored. The purpose of our study was to guide the postoperative management of this group of women.

## Methods

### Clinical data

Case inclusion criteria were women of child-bearing age who had been diagnosed with HSIL by biopsy, including CIN3 and part of CIN2. All women were premenopausal women. Case exclusion criteria were women with complications, such as endometrial carcinomas; cervical cancer including micro-invasion; incomplete information, such as lacking correlations among cytology, biopsy, and colposcopic findings; and no contact information.

**Fig. 1:**
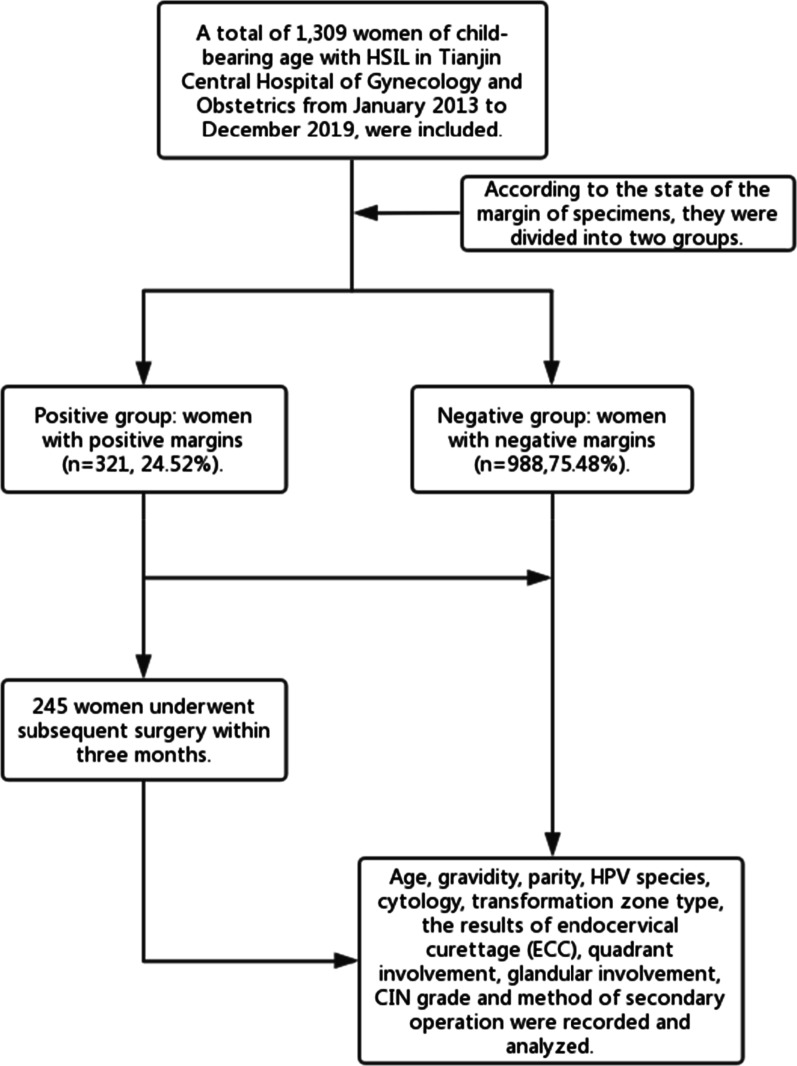
Flow chart

A total of 1309 women of child-bearing age with HSIL (including CIN3 and part of CIN2) in Tianjin Central Hospital of Gynecology and Obstetrics from January 2013 to December 2019, aged from 20 to 49 years old, were diagnosed with a "three-step" method, including cytology, colposcopy, and histology [8]. All women were premenopausal women. And all women underwent CKC. According to the state of the margin of specimens, they were divided into two groups: (1) positive group: women with positive margins (321, 24.52%); and (2) negative group: women with negative margins (988, 75.48%). Among them, 245 women underwent subsequent surgery within three months. Age, gravidity, parity, HPV species, cytology, transformation zone type, the results of endocervical curettage (ECC), quadrant involvement, glandular involvement, and CIN grade were recorded (Fig. 1). The data were collected from the electronic medical records of the institution while preserving patient anonymity. The research ethics committee waived the requirement for ethical approval and informed consent because the study used previously stored data.

### Criteria for positive margins and residual lesions

If HSIL were found in the resection margin of about 1 mm or less, including the ectocervical margin, endocervical margin, or both, it was regarded as a positive margin. If HSIL were diagnosed in women who underwent secondary surgery within three months, it was assumed to be a residual lesion. CIN1 was not considered to be a residual lesion in this study.

### Statistical methods

SPSS21.0 software was used for statistical analysis (SPSS Inc, Chicago, IL, USA). The statistical methods were examination of exact probabilities in a fourfold table and chi-squared tests. Multivariate analysis was performed using a logistic regression model. All tests were two-sided, and the level of significance was set at *P* < 0.05.

## Results

The average age of women was 38 ± 7.1 (range 20–49) years. According to the state of the margin of specimens, they were divided into two groups: (1) positive group: women with positive margins (321, 24.52%); and (2) negative group: women with negative margins (988, 75.48%). In women with positive margins, 245 cases underwent subsequent surgery within three months, including secondary CKC, LEEP, and total hysterectomy.

### Association between clinicopathological factors and positive margins

Women in both groups were divided into two levels, depending on age gravidity, parity, HPV species, cytology, transformation zone type, the results of ECC, quadrant involvement, glandular involvement, and CIN grade. There was no significant difference in age, gravidity, and parity between the two groups (*P* > 0.3). The high-risk HPV (HR-HPV) infection rate in the positive group was significantly higher than in the negative group (*P* < 0.001). The preoperative cytology results in the positive group were mainly HSIL and high-grade squamous intraepithelial lesions (ASC-H). These results were significantly different from that in the negative group (*P* < 0.001).In the negative group, the preoperative cytology results were mainly intraepithelial lesion or malignancy (NILM) and atypical squamous cells undetermined significance (ASCUS). The primary type of transformation zone in the positive group was type III (61.08%), while the proportion of type III in the negative group was only 38.92%.There was a significant difference in the transformation zone type between the two groups (*P* < 0.001). There was also a significant difference in the results of ECC between the two groups (*P* = 0.002). There was no significant difference in the percentage of women with glandular involvement and CIN grade between the two groups (*P* > 0.3). Multiple quadrant involvement was more frequently found in the positive margin group, which was significantly different from that in the negative group (*P* < 0.001; Table 1).

**Table 1:** Association between clinicopathological factors and positive margins

Characteristics	Positive (n = 321)	Negative (n = 988)	Chi-squared	*P* value
*Age (years)*				
≤ 35	23.02(102/443)	76.98(341/443)	0.812	0.368
> 35	25.29(219/866)	74.71(647/866)		
*Gravidity*				
≤ 3	25.96(135/520)	74.04(385/520)	0.965	0.326
> 3	23.57(186/789)	76.43(603/789)		
*Parity*				
≤ 2	23.84(201/843)	76.16(642/843)	0.590	0.442
> 2	25.75(120/466)	74.24(346/466)		
*Cytology*				
Minor abnormalities	12.06(55/456)	87.94(401/456)	58.707	< 0.001
Major abnormalities	31.18(266/853)	68.82((587/853)		
*High-risk HPV*				
Yes	31.42(268/853)	68.58(585/853)	62.912	< 0.001
No	11.62(53/456)	88.38(403/456)		
*Transformation zone*				
I and II	11.08(106/957)	88.92(851/957)	347.6	< 0.001
III	61.08(215/352)	38.92(137/352)		
*ECC*				
Positive	39.50(188/476)	60.50(288/476)	9.594	0.002
Negative	15.97(133/833)	84.03(700/833)		
*Quadrant involvement*				
Single	12.01(73/608)	87.99(535/608)	96.088	< 0.001
Multiple	35.38(248/701)	64.62(453/701)		
*Glandular involvement*				
Yes	23.83(194/814)	76.17(620/814)	0.533	0.457
No	25.66(127/495)	74.34(368/495)		
*CIN grade*				
CIN2	26.42(79/299)	73.58(220/299)	0.755	0.385
CIN3	23.96(242/1010)	76.04(768/1010)		

Minor abnormalities included NILM (negative for intraepithelial lesion or malignancy), ASCUS (atypical squamous cells of undetermined significance), and LSIL (low-grade squamous intraepithelial lesion); major abnormalities included ASC-H (atypical squamous cells, cannot exclude high-grade squamous intraepithelial lesion) and HSIL (high-grade squamous intraepithelial lesion)

### Logistic regression analysis of the risk factors for positive margins

In order to evaluate which variables could be considered as independent predictors for positive margins after conization, we used Logistic regression analysis. It was found that major cytology abnormality, HR-HPV infection, type III transformation zone, positive ECC result, and multiple quadrant involvement were independent risk factors for the positive margins (*P* < 0.02; Table 2).

**Table 2:** Logistic regression analysis of the risk factors for positive margins

Variables	B	SE	Wald	*P* value	OR	OR (95% CI)
Cytology	1. 161	0. 427	13.879	< 0. 001	2.614	2.241–4.249
High-risk HPV	1.198	0.254	15. 129	< 0. 001	3. 612	2.388–5.997
Transformation zone	0. 912	0. 138	8.268	0. 001	1.825	1.675–3.111
ECC	0. 685	0.612	5.308	0.008	1.744	1.187–3.146
Quadrant involvement	1. 032	0. 423	5.714	0. 011	1.620	1.366–2.124

### Univariate and multivariate analyses of risk factors for residual lesions in women with positive margins

In women with positive margins, 245 cases underwent subsequent surgery within three months, and of them, 93 cases received hysterectomy, 152 cases chose secondary conization (70 cases in LEEP and 82 cases in CKC). 83 cases (33.88%) had residual lesions detected in the postoperative specimens. Univariate analysis showed that age > 35 years, the severity of cytology results, high-risk HPV infection, type of transformation zone, the ECC result, and quadrant involvement were associated with residual lesions of the women with positive margins after CKC (*P* < 0.05). In a multivariate analysis, age > 35 years, a major cytology abnormality (including HSIL and ASC-H), high-risk HPV infection, type III transformation zone, positive ECC result, and multiple quadrant involvement were all risk factors for residual lesions (*P* < 0.05; Table 3).

**Table 3:** Univariate and multivariate analysis of risk factors for residual lesions in women with positive margins

Variable	Residual rate (%)	Univariate	Multivariate	
		*P* value	OR (95%CI)	*P* value
*Age(years)*				
≤ 35	24.39 (20/82)	0.026	1.429 (1.056–2.968)	0.037
> 35	38.65 (63/163)			
*Gravidity*				
≤ 3	33.70(31/92)	0.507	Variable removed	
> 3	33.99(52/153)			
*Parity*				
≤ 2	30.30(40/132)	0.428	Variable removed	
> 2	38.05(43/113)			
*Cytology*				
Minor abnormalities	15.19(12/79)	< 0.001	3.143 (1.986–5.113)	< 0.001
Major abnormalities	42.77(71/166)			
*High-risk HPV*				
Yes	44.59(33/74)	0.020	1.483 (1.345–3.226)	0.029
No	29.24(50/171)			
*Transformation zone*				
I and II	25.32(39/154)	< 0.001	2.996 (1.636–4.825)	0.001
III	48.35(44/91)			
*ECC*				
Positive	45.88(39/85)	0.005	2.127 (1.118–2.970)	0.007
Negative	27.50(44/160)			
*Quadrant involvement*				
Single	25.19(33/131)	0.003	1.824 (1.441–2.609)	0.004
Multiple	43.86(50/114)			
*Glandular involvement*				
No	32.82(43/131)	0.636	Variable removed	
Yes	35.09(40/114)			
*CIN grade*				
CIN2	30.19(32/106)	0.251	Variable removed	
CIN3	36.69(51/139)			
*Site of margin involvement*				
Endo	33.33(25/75)	0.866	Variable removed	
Ecto	32.35(33/102)			
Endo/Ecto	36.76(25/68)			
*Method of secondary operation*				
CKC	29.27(24/82)	0.349	Variable removed	
Total hysterectomy	38.70(36/93)			
LEEP	32.86(23/70)			

## Discussion

Cervical conization is the preferred method for the diagnosis and treatment of CIN. CKC is one type of cervical conization. Because CIN lesions are often multipoint in distribution, residual lesions and positive margins are inevitable. Studies have indicated that the incidence of residual lesions in women with positive margins after cervical conization was higher than that in women with negative margins, and the recurrence rate of women with positive margins was also higher in women with positive margins [9, 10]. It has been reported in the literature that the incidence of positive margins after cervical conization was 14–25% [2, 9, 10].which was similar to our study.However, the rate of residual lesions (33.38%) in our study was higher than in a previous study [11, 12].This may be due to the clinical characteristics of the population who chose subsequent surgeries. In recent years, the onset age of this disease has been increasingly younger. However, there is no unified clinical opinion on the risk factors and further treatment for the women of child-bearing age with positive margins. In women with no fertility requirements, considering the possibility of a poor prognosis, total hysterectomy may be possible. However, for women of child-bearing age who have fertility requirements or want to retain the uterus, it is almost impossible to accept the uterus being removed. Even secondary conization can affect conception and lead to adverse pregnancy outcomes [6, 7].Therefore, avoiding a positive margin and residual lesions, and reducing unnecessary secondary surgery are particularly important for women of childbearing age.

The results of previous studies have suggested that the possible risk factors for residual lesions after cervical conization mainly include positive margins of the specimen, involvement of the lateral margin of the cervical canal, involvement of the lateral margin of the cervical canal and outer orifice, positive specimen from ECC, menopause, persistent infection of high-risk HPV after cervical conization, and decreased or suppressed immune function [13, 14]. Many factors affect the condition of the margin after CKC, including age, menopausal status, glandular involvement, smoking, infection, training level of the operator, and other factors [15, 16]. In this study, the clinicopathological characteristics of 1309 women of child-bearing age after initial CKC and 245 cases of women with positive margins who accepted subsequent surgery within three months were retrospectively analyzed. It was found that a major cytology abnormality (including HSIL and ASC-H), HR-HPV infection type III transformation zone, positive ECC result, and multiple quadrant involvement were the common risk factors for positive margins and residual lesions. Age > 35 years was also a risk factor for residual lesions in women with positive margins after initial CKC.

Whether the severity of a cytological abnormality is related to positive margins has been long disputed by researchers. Ryu A reported that the cytologic grade before cervical conization was not a risk factor for residual disease or recurrence [17]. However, Ayhan showed that the result of a smear was an advantageous predictor for a positive ectocervical margin, and it was associated with a decrease in the occurrence rate of positive margins and residual lesions [18]. In this study, the women with major cytology abnormalities (including ASC-Hs and HSIL) were more likely to present with positive margins and residual lesions than women with minor cytology abnormalities (including NILM, ASCUS, and LSIL) before CKC,which was consistent with previous researches [17, 18]. It was found that the severity of cytology before conization was a risk factor for positive margins and residual lesions in this study. With an increase in the cytological grade, CIN levels increased, which meant that the depth of the lesion cells occupying the squamous epithelium increased, and the possibility of positive margins and residual lesions increased.

High-risk HPV infection has been recognized as a necessary condition for the occurrence and development of cervical squamous epithelial lesions and cervical cancer [19]. In this study, the rate of high-risk HPV infection in women with positive margins after CKC was 83.50% (268/321). Multivariate analysis showed that a high-risk HPV infection was the independent risk factor for positive margins and residual lesions, which was consistent with previous study [20, 21]. However, at present, the pathological mechanism for a high rate of positive margins and residual lesions after cervical conization caused by high-risk HPV infection is not very clear; it may due to the cervical lesion area in women with CIN infected by high-risk HPV was larger and deeper than that of women infected by low-risk HPV [22]. Giorgio Bogani also reported that HR-HPV-negative high-grade cervical dysplasia experience more favorable outcomes than patients with documented HR-HPV infection(s) [23].

Further study is needed. Based on the above studies, the HPV type can better predict postoperative positive margins and residual lesions.

In addition, it was also found that women with a type III transformation zone were more likely to have positive margins and residual lesions after an operation. This may be due to the lesion invading the cervical tube. CKC cannot completely remove diseased tissue. In the same way, the rates for positive margins and residual lesions in women with positive results of ECC were higher than that of negative results. The same results were found in previous studies [23, 24]. Researchers also found that multiple quadrant involvement was a risk factor for positive margins and residual lesions [25, 26]. It came to the same conclusion based on our foundings. In this study, the rate of multiple quadrant involvement was 77.26% in the women with positive margins, and that in the women with negative margins was 45.85%. Increases in the range of lesions likely affected observations during the operation, interfered with the judgment of surgical margins, and increased the difficulty of surgery. The above factors should be taken into account in the process of cervical conization to achieve the goal of leaving no residual lesions and preserving cervical function to a greater extent, that is, more attention should be paid to the extent and depth of lesions removed by cervical conization.

There was no significant difference between women of age > 35 years and ≤ 35 years in the rate of positive margins, but there was a significant difference in the rate of residual lesions between the two age groups. The rate of residual lesions in the age > 35 years group was higher than the ≤ 35 years group (*P* < 0.03). This may be due to the persistent infection of HPV, especially high-risk HPV. Sarian reported that women older than 35 years had a significantly higher risk for persistent infection following LEEP [27]. This could cause multiple quadrant lesions of the cervix [22]. Furthermore, the older the patient, the higher the degree of cervical atrophy. The cervical transformation zone and lesions therefore moved inward to the cervical canal, so cervical conization could not completely remove the diseased tissue. Bilibio also considered that increasing age was the only factor that accurately predicted residual disease [15]. All of these results indicated older age was a predictive factor for residual lesions and it can play a better role in guiding the formulation of a postoperative treatment plan for women of child-bearing age with positive margins. Subsequently, normal reproductive function and organ integrity can be preserved as much as possible in women younger than 35 years old.

## Conclusion

Our study's main strength was the particular group of patients we included: women of child-bearing age. This group of patients has the strongest desire to preserve reproductive function or uterus and the postoperative treatment of positive margins after CKC is faced with more challenges. Besides, patients with positive margins who underwent the secondary surgery were from the same sample group, reducing bias and achieving more accurate results.

However, this study also had unavoidable limitations of its retrospective design. First, we could not assess all variables potentially associated with residual lesions in the single study. Furthermore, the cases involving only one hospital might have reduced our results' external validity, and further prospective studies with a larger sample size in a broader context are needed. Additionally, the women in our cohort were tested for HPV and TCT, which largely determined subsequent treatment. However, from the analysis results of residual lesions, it could be seen that there are still a large number of people who can avoid secondary surgery, which requires more accurate biomarkers to reduce the rate of secondary surgery [28–30].

In conclusion, a major cytology abnormality (including HSIL and ASC-H), HR-HPV infection, III transformation zone, positive ECC result, and multiple quadrant involvement were the common risk factors for positive margins and residual lesions. Age > 35 years was also a risk factor for residual lesions in women with positive margins after initial CKC. Experienced doctors should treat these high-risk women appropriately, operate prudently, and expand the scope of the operation while considering the patient’s fertility requirements. Furthermore, the patient with positive margins after CKC should be managed individually. It is reasonable to require further surgical treatment in women without fertility requirement, and close follow-up is necessary for women who have fertility requirements or are unwilling to undergo subsequent surgery if they have no risk factor, especially for women < 35 years.

## Data Availability

Not applicable.

## Abbreviations

HSIL: High grade cervical intraepithelial lesions
HPV: Human papillomavirus
HR-HPV: High-risk human papillomavirus
CIN: Cervical intraepithelial neoplasia
CKC: Cold knife conization
ECC: Endocervical curettage
TCT: Thin prep cytologic test
LEEP: Loop electrosurgical excisional procedure
NILM: Negative for intraepithelial lesion or malignancy
LSIL: Low grade cervical intraepithelial lesions
HSIL: High grade cervical intraepithelial lesions
ASCUS: Atypical squamous cells of undetermined significance
ASC-H: Atypical squamous cells, cannot exclude high-grade squamous intraepithelial lesion
