# Correction to: Necessity for subsequent surgery in women of child-bearing age with positive margins after conization

**DOI:** 10.1186/s12905-021-01367-5

**Published:** 2021-06-01

**Authors:** Xinmei Wang, Juan Xu, Yang Gao, Pengpeng Qu

**Affiliations:** 1 Clinical College of Central Gynecology and Obstetrics, Tianjin Medical University, Tianjin, 300070 China; 2 Department of Gynecological Oncology, Tianjin Central Hospital of Gynecology and Obstetrics, Tianjin, 300100 China

## Correction to: BMC Women’s Health (2021) 21:191 https://doi.org/10.1186/s12905-021-01329-x

Following the publication of the original article [1], we were notified that Pengpeng Qu should be marked as corresponding author.

The original article has been corrected.
